# Rapid exposure of macrophages to drugs resolves four classes of effects on the leading edge sensory pseudopod: Non-perturbing, adaptive, disruptive, and activating

**DOI:** 10.1371/journal.pone.0233012

**Published:** 2020-05-29

**Authors:** Thomas C. Buckles, Brian P. Ziemba, Danijel Djukovic, Joseph J. Falke

**Affiliations:** Department of Biochemistry, and Molecular Biophysics Program, University of Colorado Boulder, Boulder, Colorado, United States America; Universidade Federal de Juiz de Fora, BRAZIL

## Abstract

Leukocyte migration is controlled by a membrane-based chemosensory pathway on the leading edge pseudopod that guides cell movement up attractant gradients during the innate immune and inflammatory responses. This study employed single cell and population imaging to investigate drug-induced perturbations of leading edge pseudopod morphology in cultured, polarized RAW macrophages. The drugs tested included representative therapeutics (acetylsalicylic acid, diclofenac, ibuprofen, acetaminophen) as well as control drugs (PDGF, Gö6976, wortmannin). Notably, slow addition of any of the four therapeutics to cultured macrophages, mimicking the slowly increasing plasma concentration reported for standard oral dosage in patients, yielded no detectable change in pseudopod morphology. This finding is consistent with the well established clinical safety of these drugs. However, rapid drug addition to cultured macrophages revealed four distinct classes of effects on the leading edge pseudopod: (i) non-perturbing drug exposures yielded no detectable change in pseudopod morphology (acetylsalicylic acid, diclofenac); (ii) adaptive exposures yielded temporary collapse of the extended pseudopod and its signature PI(3,4,5)P_3_ lipid signal followed by slow recovery of extended pseudopod morphology (ibuprofen, acetaminophen); (iii) disruptive exposures yielded long-term pseudopod collapse (Gö6976, wortmannin); and (iv) activating exposures yielded pseudopod expansion (PDGF). The novel observation of adaptive exposures leads us to hypothesize that rapid addition of an adaptive drug overwhelms an intrinsic or extrinsic adaptation system yielding temporary collapse followed by adaptive recovery, while slow addition enables gradual adaptation to counteract the drug perturbation in real time. Overall, the results illustrate an approach that may help identify therapeutic drugs that temporarily inhibit the leading edge pseudopod during extreme inflammation events, and toxic drugs that yield long term inhibition of the pseudopod with negative consequences for innate immunity. Future studies are needed to elucidate the mechanisms of drug-induced pseudopod collapse, as well as the mechanisms of adaptation and recovery following some inhibitory drug exposures.

## Introduction

Leukocytes, including macrophages and neutrophils, possess a sophisticated chemosensory system that controls cellular migration up primary attractant gradients to sites of infection, injury, or tumor formation during the innate immune response [1–8]. At the target site, recruited leukocytes and other cells can release secondary attractants that recruit additional leukocytes, but excessive secondary attractant signaling and recruitment may lead to local inflammation and, in extreme cases, toxic effects (reviewed in [9–14]).

The chemosensory pathway that directs leukocyte migration up primary and secondary attractant gradients is localized on the broad sensory pseudopod at the leading edge of the polarized cell, where pathway components assemble on the cytoplasmic leaflet of the plasma membrane (reviewed in [3–5, 9, 15–17]). The leukocyte chemosensory pathway is highly specialized, conferring unique properties to leukocyte chemotaxis not observed in other cell types. In particular, the leukocyte pathway possesses a positive feedback loop that is able to maintain the stability and ruffling activity of the leading edge pseudopod even in the absence of an attractant gradient, enabling the pseudopod to rapidly detect and direct migration up a newly appearing gradient [3, 4, 16, 18–26]. The feedback loop is comprised of multiple essential signaling proteins, cytoskeletal elements, and second messengers, including: phosphoinositide-3-kinase (PI3K); protein kinase C (PKC); small G-proteins (Ras, Rac); actin filaments; the signaling lipid phosphatidylinositol-3,4,5-trisphosphate (PIP_3_); and Ca^2+^ ions [2, 7, 16, 18, 19, 21–24, 26–40]. Activation of any loop component leads to a characteristic co-activation of other loop components, increased activity of the lipid kinase PI3K and accumulation of its product PIP_3_ signaling lipid, and expansion of the leading edge pseudopod. By contrast, inhibition of any loop component yields inactivation of other loop components, decreased PI3K activity and PIP_3_ levels, and contraction of the pseudopod.

The leading edge chemosensory pathway also possesses an intrinsic adaptation mechanism that enables the leading edge pseudopod to adapt as needed to changes in its chemical environment. Adaptation is a ubiquitous feature of chemosensory pathways in archaea, bacteria, and eukaryotes [41–45]. Such intrinsic adaptation adjusts pathway gain and dampens background signals, thereby preventing signal saturation and maintaining sensory function as the cell migrates up an attractant gradient in a complex chemical environment with competing stimuli. In leukocytes, intrinsic pseudopod adaptation is provided by a hardwired adaptation branch of the chemosensory pathway that regulates the net activity of specific pathway components, often by covalent modification [41–45]. Different types of leukocytes, such as macrophages and neutrophils, possess different classes of cell surface receptors and other specializations, but appear to share similar mechanisms of chemosensing and intrinsic adaptation [15, 20, 32, 46]. Such intrinsic pseudopod adaptation could in principle counteract perturbations triggered by drug exposure, thereby restoring normal pseudopod morphology and function. Alternatively, extrinsic intracellular or extracellular processes besides intrinsic chemosensory adaptation could counteract drug-induced perturbations; such extrinsic mechanisms could extrude, sequester, or chemically modify a drug that perturbs the leading edge pseudopod [47–49].

The leading edge pseudopod is clearly visualized in live, cultured leukocytes by differential interference contrast microscopy (DICM) or by fluorescence microscopy [26, 30]. Prior studies have shown that drugs that target key components of the leading edge positive feedback loop yield dramatic changes in the size of the pseudopod and its leading edge PIP_3_ signal [26, 30, 50–54]. Well characterized inhibitors of the leading edge positive feedback loop that collapse the leading edge pseudopod include wortmannin, a covalent inhibitor of PI3K that directly inhibits leading edge PIP_3_ production, and Gö6976, an inhibitor of PKC protein kinase activity that indirectly inhibits PIP_3_ production by blocking a key component of the feedback loop [26]. Natural attractants for leukocytes include platelet-derived growth factor (PDGF), a powerful macrophage attractant that activates a receptor tyrosine kinase (RTK, in particular the PDGF receptor) that stimulates PI3K and PIP_3_ production. PDGF has been observed to expand the leading edge pseudopod, increase its PIP_3_ signal, and drive increased levels of cell polarization [26, 55].

The present study begins by developing a novel combination of live cell assays and applying them to cultured, polarized RAW macrophages in order to quantify the effects of four therapeutic and three control drugs on leading edge pseudopod morphology. The four selected therapeutic drugs possess well established clinical safety and are investigated within their standard clinical dosages (i.e. total plasma concentrations, [56–62]). Three are non-steroidal anti-inflammatory drugs (NSAIDs) that inhibit cyclooxygenase enzymes (COX) and thereby inhibit the production of inflammatory signals [63–65]: acetylsalicylic acid (aspirin), ibuprofen and diclofenac. The fourth, acetaminophen (paracetamol), is not an NSAID and relieves pain via unknown mechanisms. The three control drugs, wortmannin, Gö6976, and PDGF each target a known pathway component (see above) and are employed at their standard experimental concentrations [26, 30, 50–54].

The findings demonstrate that the present approach can resolve drug effects on the leukocyte leading edge pseudopod of polarized, cultured macrophages into four operational classes: (i) non-perturbing exposures, (ii) adaptive exposures, (iii) disruptive exposures, and (iv) activating exposures. When any of the four therapeutic drugs are added slowly to mimic the slow rise of drug levels in the plasma of patients following standard oral dosages [56–62, 66], these drugs are found to have little or no effect on leading edge morphology, as consistent with their well-established clinical safety. However, rapid addition of the therapeutic and control drugs reveals the four operational classes. Acetylsalicylic acid and diclofenac are classified non-perturbing because they have little or no detected effect on the macrophage leading edge pseudopod and its PIP_3_ signal. For these drugs, either rapid or slow addition retains normal pseudopod morphology in single cell and population studies. In contrast, ibuprofen and acetaminophen are classified as adaptive because rapid addition of either drug to polarized cells yields short-term collapse of the leading edge pseudopod and loss of the PIP_3_ signal, followed by slow recovery. In contrast to the four therapeutic adaptive drugs, the two non-clinical control inhibitors wortmannin and Gö6976 are each known to directly inhibit key components of the leading edge positive feedback loop and rapid addition is observed herein to trigger long term collapse of the pseudopod with no detected recovery as previously observed [26, 30, 50–54]. At the other extreme, PDGF is a well studied attractant and rapid addition yields leading edge expansion and increased cell polarization over the three hour observation period as predicted [26, 55]. Overall, the findings provide impetus for future studies probing the inhibitory mechanisms underlying therapeutic drug-triggered pseudopod collapse, as well as the adaptive mechanisms that enable pseudopod recovery following rapid exposure to some perturbing drugs.

## Methods

### Reagents

RAW 264.7 macrophages were obtained from the American Type Culture Collection (ATCC TIB-71, Manassas, VA; Lots 70000171, 70012232 with cell identity confirmed by ATCC via growth properties, morphology, and COI assay). Cell media (supplemented DMEM) was composed of DMEM obtained from Thermo Fischer Scientific (Waltham, MA), fetal bovine serum (FBS, contains ~400 μM BSA as specified by manufacturer) from Millipore-Sigma (St. Louis, MO), penicillin, streptomycin and GlutaminePlus (L-alanyl-L-glutamine) from Atlanta Biologicals (Flowery Branch, GA). Dulbelcco's phosphate buffered saline (D-PBS) was from Gibco (Gaithersburg, MD). All cell media was regularly subjected to mycoplasma testing to ensure lack of infection. Matriplate 96 well imaging plates were from Matrical, Inc. (Spokane, WA). Acetylsalicylic acid (Lot SLBV2290), ibuprofen (Lot SLBX6584), acetaminophen (Lot SLBR2060V), diclofenac (Lot BCBS5961V), wortmannin (Lot 086M4026V), PDGF (Lot SLB63548V), and HEPES (free acid) were obtained from Millipore-Sigma (St. Louis, MO). Gö6976 (Lot 6A/174381) was purchased from Tocris Biosciences (Minneapolis, MN). CellMask Deep Red and Green membrane stains were purchased from ThermoFischer Scientific (Waltham, MA). Cell-culture grade DMSO was purchased from MP Biomedicals (Santa Ana, CA). AKT-PH-mRFP mammalian expression plasmid was subcloned by John H. Evans [26, 30].

### Cell culture and transfections

Cell culture techniques used herein have been previously described [26, 30]. Briefly, starting from cryogenic stocks, RAW macrophages were grown as per ATCC recommendation at 37˚C under 5% CO_2_ in supplemented DMEM media containing 2 mM GlutaminePlus, 20 mM HEPES pH 7.2 with NaOH, 100 U/mL penicillin, 100 μg/mL streptomycin, and supplemented with 10% FBS (where undiluted FBS contains ~400 μM BSA as specified by manufacturer), resulting in a final albumin concentration of ~40 μM. Cells were washed in D-PBS pH 7.2 with HCl and passaged upon reaching ~80% confluency, as recommended by ATCC. Single cell or population experiments used RAW cells following at most 14 or 5 such passages, respectively. For studies of cells transfected with AKT-PH-mRFP to monitor PIP_3_ lipid, the transfection protocol using the Neon electroporation system has been previously described [26].

### Microscopy

**Fig 1:**
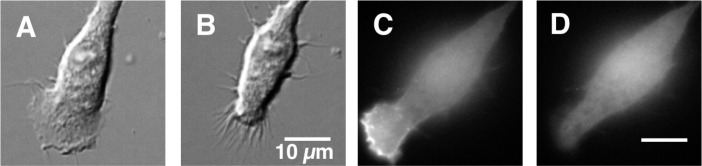
Imaging highly polarized, actively ruffling cultured RAW macrophages and drug-induced collapse of the leading edge pseudopod. RAW macrophages were plated on glass at 37°C in the absence of an attractant gradient. Individual, spontaneously polarized cells exhibiting extended, ruffling, leading edge pseudopods were imaged as described in Methods. These cells migrate slowly on glass and are thus well suited for quantitative imaging of event timecourses at their leading edges. Drugs were added at t = 0 to the total concentration indicated in Fig 2. *Panels A through D* images show representative effects of drug addition on the leading edge pseudopod: (A,B) DICM images of a single cell at t = 0 and 5 min after ibuprofen addition, respectively, illustrating the drug-triggered loss of leading edge pseudopod area; (C,D) fluorescence images of the PIP_3_ sensor AKTPH-mRFP in a single cell at t = 0 and 5 min after wortmannin addition, respectively [26, 30] illustrating the drug-triggered loss of the signaling lipid PIP_3_ on the leading edge pseudopod.

Live-cell microscopy methods have been previously described [26]. Cells were counted by hemocytometer and plated at known density in 96 well imaging plates, then subsequently incubated at 37˚C under 5% CO_2_ for ~24 hours to facilitate cell adhesion and generate conditions of steady state cell polarization. In the bottom of each well, cells attach to a flat glass surface and many spontaneously polarize in the absence of an attractant gradient. Two microscopes were employed. (i) Images for Figs 1A and 1B and 4C and 4D and S1 and S3 were captured using a Nikon TiE microscope equipped with a 40x, 0.95 N.A. objective and a Hamamatsu ORCA-Flash 4.0 V3 Digital CMOS camera. (ii) Images for Figs 1C and 1D and 4E–4L and S2 were acquired with a Nikon TiE spinning-disc confocal microscope equipped with a Yokogawa CSU-X1 scanning unit, an Andor iXon 888 EMCCD camera, and a 60x, 1.3 N.A. water-immersion objective (Fig 1C and 1D) or a 40x, 0.95 N.A. objective (Figs 4E–4L and S2). For both microscopes, the imaging stage was enclosed in an environmental chamber maintaining humidity, 5% CO_2_, and 37°C.

### Drug addition and mixing

Imaging studies monitored a single cell, or a population of cells, following the addition of drug or its carrier as a control. Two types of addition protocols were employed. In rapid addition the drug or carrier was added (0.5 μL into 500 μL) at t = 0 via a 2 μL micropipettor (Gilson) with immediate, gentle pump-mixing with the same micropipettor and tip to yield the final concentration within 10 sec. This pump-mixing procedure slowly drew and expelled 100 μL of media into and out of the tip, and then repeated for a total of 5 pump-mixing cycles during the 10 sec mixing time. In slow addition the drug or carrier was added via a stepwise procedure over a period of 90 min to generate a more gradual rise up to the same final total drug concentration as rapid addition. This slow addition protocol added the drug in four equal increments using a 1 μL pre-calibrated Hamilton syringe (each 0.25 μL into 500 μL) at t = 0, 30, 60, 90 min with immediate, gentle pump-mixing as above to generate a more gradual, step-wise increase in concentration. Final total drug concentrations were PDGF ββ 2.1 μM, wortmannin 500 nM, Gö6976 1.0 μM, acetylsalicylic acid 1.1 mM, diclofenac 17 μM, ibuprofen 240 μM, acetaminophen 170 μM. All drugs were added in carrier DMSO with the exception of PDGF ββ, which was added in supplemented DMEM.

Care was taken to ensure that drug effects observed after addition were due to the intended global increase in the total drug concentration, not due to locally high drug concentrations prior to complete mixing. The microscopic field observed following drug addition in single cell and population measurements represented a small fraction (< 0.2% and < 6%, respectively) of the 8 x 8 mm^2^ glass surface area in the observation well. In single cell and population studies, cells appropriate for imaging were selected at random locations in three of the four quadrants of the surface, then drug was added at the outside corner of the remaining quadrant (always the outside corner of the far-left quadrant) followed by pump mixing at that same location. No correlation was observed between drug effects on cells and the distance of the cells from the drug addition. Moreover, in single cell measurements each cell observed exhibited approximately the same drug effect, while in population measurements all four quadrants of the large grid showed approximately the same drug effects. Such independence of drug effects from the distance to the point of addition indicated the effects were global, not local.

### Timecourse measurement and error analysis

A novel cell population analysis was developed to quantify both initial and long-term drug effects, including pseudopod adaptation and recovery following an initial drug-induced perturbation. Timecourses of cell populations were measured in a large field containing 1200 ± 100 cells (Figs 3, 4A and 4B and S1), which was scanned as either 8 x 8 (Fig 4AB) or 6 x 6 (Fig 3) unit areas. The unit areas, which served as individual data points, were stitched together into a full field super-image using Nikon Elements software. For each full field, an initial super-image was captured at t = 0, then subsequent super-images were captured at the specified timepoints after drug or control addition. Analysis of population timecourse data was as follows. At each timepoint, the number of polarized cells with extended leading edge pseudopods was counted in Fiji (ImageJ) yielding an average over the 64 (Fig 4AB) or 36 (Fig 3) unit areas of that super-image. For each super-image, the resulting mean timepoints were normalized to the mean initial value of the t = 0 timepoint. Finally, the final global mean timecourse was determined by averaging the corresponding mean normalized timepoints from different super-images (experiments), yielding the global mean timepoints and their SEM for N = the number of super-images. The resulting global mean timecourses represent averages over 3–6 super-images (experiments) each containing approximately 1000 cells and collected from independent wells over 3–6 separate days for therapeutic drug treatments, or 2–6 separate days for control treatments.

Single-cell timecourses (Fig 5) were measured and analyzed as previously described for individual, polarized cells with extended, actively ruffling leading edge pseudopods [26]. Briefly, for each cell the value of the initial t = 0 timepoint was used to normalize all timepoints. Subsequently, for a group of at least 3 cells in an experiment carried out on the same day, corresponding normalized timepoints were averaged to generate a mean value for each timepoint. Finally, the resulting mean timepoints for individual experiments were averaged over all experiments to generate a global mean and its standard error of the mean (SEM) for N = the number of experiments. Final global averages represented 17–32 total cells imaged in at least 3 experiments on at least 3 separate days for experimental treatments, or 9–15 total cells imaged in at least 3 experiments on at least 2 separate days for control treatments.

### Liquid chromatography-mass spectrometry sample preparation

Cell cultures were prepared for mass spectrometry analysis of drug stability using exactly the same procedures described above for long-term studies of drug effects on cell populations (above). 50 μL of media were taken from the culture immediately after drug addition and mixing, then snap frozen in LN_2_. Cultures were allowed to incubate 180 minutes before another 50 μL sample was withdrawn and snap frozen. Images of the cell population in each culture were analyzed to ensure the drug effects on the cells were within their characteristic ranges. The number of replicate samples, each prepared from separate cultures, ranged from 3 (acetylsalicylic acid and diclofenac) to 6 (acetaminophen and ibuprofen). Means and standard deviations were calculated for each set of replicates.

After storage at -80°C and thawing, samples were diluted into 250 μL MeOH containing 50 μM meloxicam as an internal sample standard. Samples were then stored at -20°C for 20 minutes and subsequently spun at 15,000 x g for 15 minutes. While not disturbing the precipitated pellet, 200 μL of supernatant was extracted and dried via speedvac.

Prior to LC-MS analysis, dried study samples containing each drug were reconstituted in 200 μL (ibuprofen, acetaminophen and aspirin) or 100 μL (diclofenac) of 20 mM ammonium acetate in 60% water/35% acetonitrile/ 5% methanol + 0.1% formic acid. Except for acetaminophen samples, the reconstitution solvent also contained 20 μM 4-amino-salicylic acid (4-ASA) that was used as a post-reconstitution internal standard to monitor final sample preparation and mass spec performance (4-ASA overlapped the acetaminophen peak).

The LC system was composed of an Agilent 1100 binary pump, an Agilent 1110 auto-sampler and an Agilent 1100 temperature-controlled column compartment (Agilent Technologies, Santa Clara, CA). 10 μL of each sample was injected into the LC. Chromatography was performed in reverse phase (RP) on Agilent Eclipse XDB-C8 column (150 x 4.6 mm, 5.0 μm particle size, Agilent Technologies, Santa Clara, CA). The flow rate was 0.400 mL/min, auto-sampler temperature was kept at 4°C, and the column compartment was set at 40°C. Chromatography time was 35 min, and all 6 peaks eluted off the column between 10 and 20 min without any overlapping except for acetaminophen and 4-ASA.

### Mass spectrometry data collection and error analysis

After the chromatographic separation, MS ionization and data acquisition was performed using AB Sciex QTrap 4000 mass spectrometer (AB Sciex, Toronto, ON, Canada) equipped with electrospray ionization (ESI) source ionizing ibuprofen, aspirin, diclofenac as well as internal standards (meloxicam and 4-aminosalicylic acid) in negative and acetaminophen in positive mode, respectively. The instrument was controlled by Analyst 1.5 software (AB Sciex, Toronto, ON, Canada). Targeted data acquisition was performed in multiple-reaction-monitoring (MRM) mode. 5 MRM transitions were monitored in negative mode (4-aminosalycilic acid: 153.2 - /108.2 -; aspirin: 180.2 - /137.3 -; ibuprofen: 206.2 - /161.3 -; diclofenac: 296.2 - /250.6—and meloxicam: 351.4 - /147.0 -) whereas acetaminophen was analyzed in positive mode (MRM: 152.1 + /110 +). The source and collision gas was N 2 (99.999% purity). The ion source conditions were: curtain gas (CUR) = 25 psi, collision gas (CAD) = medium, ion spray voltage (IS) = ± 4K V, temperature (TEM) = 300°C, ion source gas 1 (GS1) = 50 psi and ion source gas 2 (GS2) = 40 psi. Each MRM transition was scanned in “unit” resolution in both Q1 and Q3.

The extracted MRM peaks were integrated using MultiQuant 2.1 software (AB Sciex, Toronto, ON, Canada). The two spiked internal standards (meloxicam and 4-aminosalicylic acid) were used to monitor sample preparation and LC-MS assay performance. A standard mixture of all four study drugs was used as a quality control to measure signal reproducibility. Intra- and inter-day reproducibility for each measured standard was under 10% without data normalization.

### Statistical tests

Replicates suitable for statistical analysis were carried out for all measurements, as described in detail above and reported in the relevant figure legend. No outliers were removed during data analysis and calculation of reported means. Data were normalized to the initial t = 0 measurement for individual cells and for populations to facilitate comparisons between different cells and different populations, respectively, given standard biological variability. Statistical significance of differences between means was calculated using Student’s unpaired t-test [67] a one-tailed test was employed for significance of a predicted increase or decrease, while a two-tailed test was used for significance of a possible difference of either sign. The threshold for significance is defined as 97% (p ≤ 0.03).

## Results

### Approach

To characterize the effects of drugs on the leukocyte leading edge chemosensory pathway, we utilized both cell population and single cell assays to monitor the leading edges of RAW macrophages plated at controlled, low density on clean glass in standard media at 37°C. Plating on clean glass significantly reduces cell movement and facilitates long-term pseudopod imaging [26, 30]. No global attractant was added (excepting experiments with global PDGF addition), and no attractant gradient was imposed. In the absence of added attractant, the leading edge positive feedback loop maintains spontaneous polarization of a sizable subset (9 ± 2%) of the macrophage population. This protocol yields large numbers of relatively immobile, easily imaged polarized cells with well-developed leading edges displaying a ruffling sensory pseudopod (see Figs 1A and 4C below) [26, 30]. Activators and inhibitors of the positive feedback loop are known to expand and contract the leading edge pseudopod, respectively, via a signaling mechanism that involves increased or decreased levels of the signaling lipid PIP_3_ in the cytoplasmic leaflet of its plasma membrane, respectively [16, 26, 30, 68]. The leading edge pseudopod also possesses an intrinsic adaptation mechanism that damps out fluctuations in the chemical environment as required for movement in an attractant gradient [42, 69, 70].

A novel cell population analysis was developed to quantify both short- and long-term effects of drugs on the leading edge pseudopods of large numbers of polarized RAW macrophages over a timescale of 2–3 hours. These studies employed DICM to record high resolution images of macrophage populations containing ~1200 total cells, including ~100 polarized cells with well-defined leading edge pseudopods, captured in subpopulations of ~20–40 total cells per unit area in a grid containing 36–64 such areas per superimage (S1 Fig). The number of cells with extended leading edge pseudopods per unit area was quantified before drug addition, and at selected timepoints after drug addition for up to 3 hours. Finally, the mean timecourses for 3–6 superimages were averaged to generate an overall mean timecourse for effect of each drug on hundreds of polarized cells across all experiments.

Single cell imaging studies were also carried out to probe the short-term effects of drugs on both the size (area) and PIP_3_ signal of the leading edge pseudopod in individual RAW macrophages. Previous studies have shown that leading edge area and PIP_3_ levels are sensitive indicators of pseudopod perturbation by drugs [26, 30, 71, 72]. Each single cell imaging experiment employed differential interference contrast microscopy (DICM) and z-stacked spinning disk confocal fluorescence microscopy to record high resolution images of the leading edge pseudopod of a polarized, actively ruffling macrophage before addition of each drug, and at selected timepoints for 5 min after drug addition. The resulting DICM images were used to quantify the timecourse of drug effects on pseudopod area, while the fluorescence images quantified levels of a standard fluorescent PIP_3_ sensor (AKT1 PH domain fused to mRFP [26, 30]) on the pseudopod membrane. For a given drug treatment, timecourses obtained from observations of 17 to 32 single cells were averaged to generate a mean timecourse for drug effects on the leading edge pseudopod area and PIP_3_ signal.

The population and single cell assays are complementary. The population analysis monitors the effects of drugs on the leading edge pseudopods of at least ~300 polarized cells over a period of hours, making it possible to quantify how drugs impact the frequency of cells with deployed, extended pseudopods, and to ascertain whether the perturbed pseudopods exhibit long term recovery following drug treatment. The single cell analysis provides a higher time-resolution view of the initial effects of drugs on the leading edge pseudopod and its PIP_3_ signal for at least 17 individual, polarized macrophages over a period of 5 minutes. In both the population and single cell assays, selective observation of polarized, ruffling cells ensures that all studied cells are healthy and possess an active leading edge pseudopod and signaling pathway. Representative cell images and drug effects are shown in Figs 1 and 4 and S2 and S3 for all drugs. Fig 1 shows representative DICM and fluorescence images of the leading edge, both before and 5 min after addition of an inhibitory drug, illustrating drug-triggered collapse of both the leading edge area (Fig 1A and 1B) and its PIP_3_ signal (Fig 1C and 1D).

**Fig 2:**
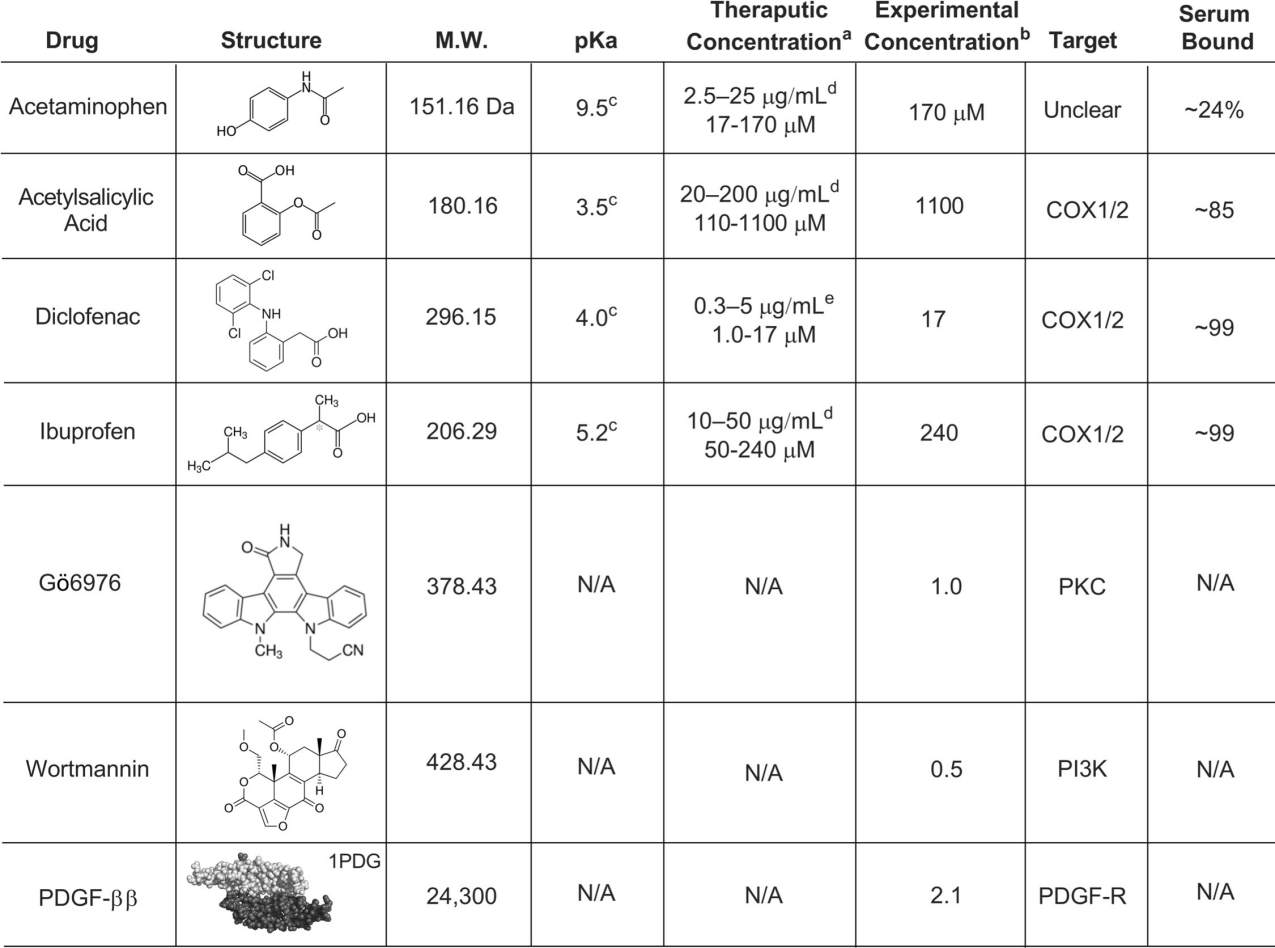
Drugs employed in the present study and key parameters. Shown are the four representative therapeutic drugs employed (acetylsalicylic acid, acetaminophen, diclofenac, ibuprofen) as well as the three control drugs (Gö6976, wortmannin, PDGF ββ). Information provided for each drug includes: chemical structure, molecular weight, pKa, therapeutic peak total concentration in human plasma following clinical dosage, total concentration employed in the present cultured cell experiments, target (where known), and percent bound to proteins in human plasma. Notes: ^a^recommended therapeutic range of total drug concentration in human plasma; ^b^experimental total drug concentration in cell culture medium (this study); ^c^references [73, 74]; ^d^references [57, 59]; ^e^reference [58]; ^f^references [75–78].

The drugs employed in the present study, their total concentrations, structures, and other key parameters are summarized in Fig 2. The four therapeutic drugs employed (acetylsalicylic acid, acetaminophen, diclofenac, ibuprofen) are each studied at the same total peak drug concentration reached in human plasma following maximum clinical oral dosage. The three control drugs employed (Gö6976, wortmannin, PDGF ββ) are each studied at their standard total concentrations in cultured cell studies [26, 30].

### Population analysis of the effects of gradual drug addition on the leading edge pseudopod

To measure drug effects on populations of polarized RAW macrophages, populations of ~1200 well-resolved cells plated on glass in standard media were imaged at 37°C in each experiment (see Methods and Approach). A target subpopulation of ~100 cells displayed a readily observed extended, ruffling leading edge pseudopod [26, 30] and were counted before drug addition, and at multiple timepoints during a 2–3 hour observation period following drug addition at t = 0. Altogether, 3 to 6 experiments were carried out for each treatment on at least 3 days, yielding average timecourses summarizing observations of ~300 to ~600 polarized cells, respectively.

**Fig 3:**
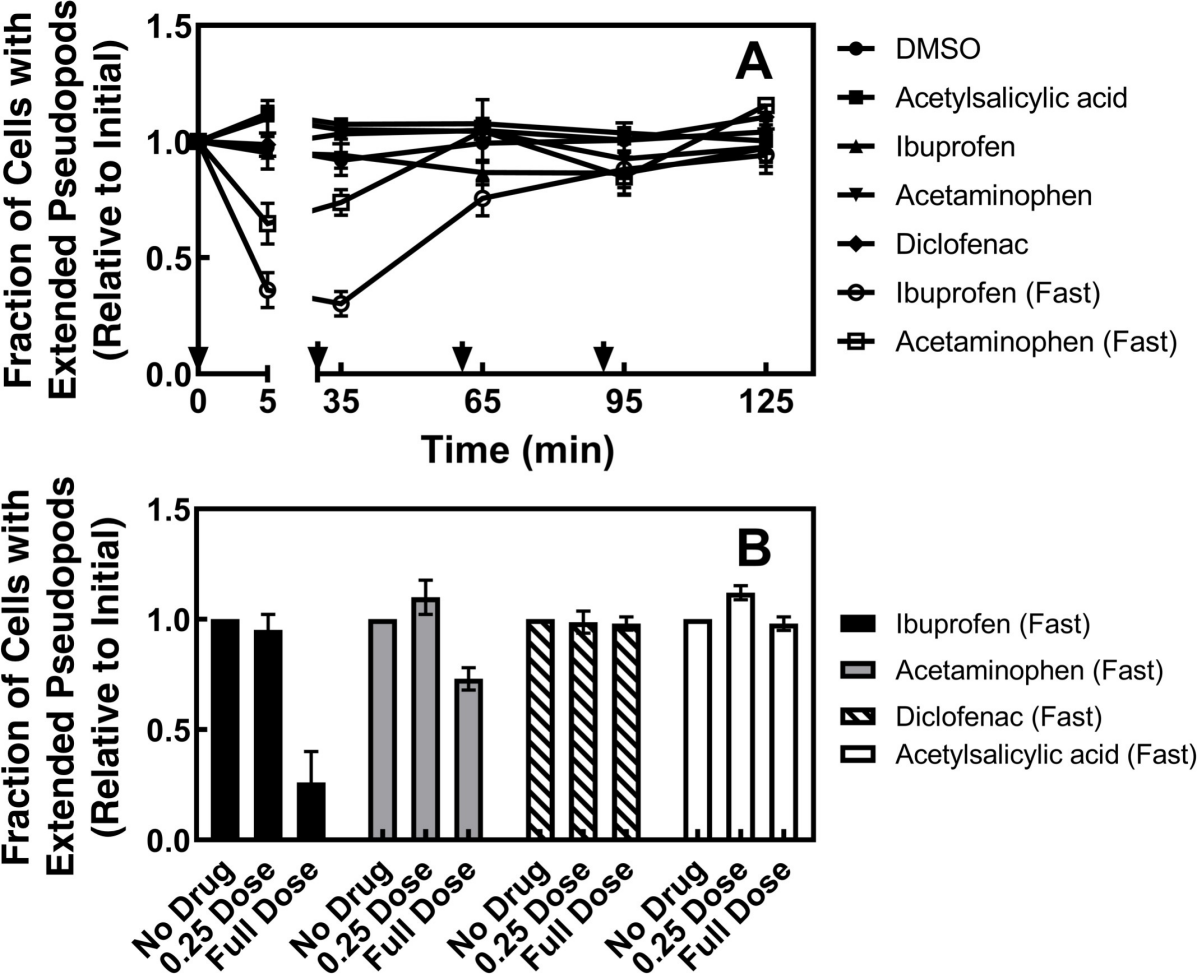
Effects of gradual drug addition on populations of polarized macrophages. RAW macrophages were plated on glass at 37°C in the absence of an attractant gradient, then images of cell populations containing large numbers of individual, spontaneously polarized cells exhibiting extended, ruffling, leading edge pseudopods were acquired as described in Methods. Subsequently, drug or carrier was added in four equal increments at 0, 30, 60, 90 min summing to yield the total drug concentration indicated in Fig 2. Images of the cell population were collected 5 min after each incremental addition at the indicated timepoints over a 2 hour observation period (filled symbols). The number of cells in the population displaying extended leading edge pseudopods was counted for each timepoint and normalized to the initial number prior to drug addition. Data collected for multiple populations was averaged, yielding the illustrated mean timecourse for each treatment. As an internal control, rapid addition at t = 0 of the full total drug concentration was also carried out for two of the four therapeutics (ibuprofen fast and acetaminophen fast, open symbols; these data can be directly compared with more extensive, independent rapid addition data in Fig 4 below). *Panel A* summarizes the resulting timecourses. *Panel B* compares the dose dependence of drug effects measured 5 min after addition at t = 0 of either 1X total drug concentration (Fig 2) or 0.25X total drug concentration. Error bars are SEM for N = 3 (fast acetaminophen) or 4 (others) experiments carried out on at least three days. Error bars smaller than their data point symbol are not visible.

Fig 3 first examines the effects of gradual addition of the four therapeutic drugs on leading edge morphology. In the clinic, these therapeutic drugs are typically administered orally, which yields gradually increasing drug levels in the plasma over a period of 30–90 min [79, 80]. Here, gradual addition of therapeutic drugs was carried out by adding four equal increments over 90 min that sum to yield the maximum total drug concentration achieved at peak plasma levels in patients (Fig 2). Fig 3A shows that such gradual, incremental addition of each of the four therapeutics or carrier has no significant effect on the number of polarized cells with extended leading edges per unit observation area (0.91 > p > 0.21 for the full range of treatments and timepoints, respectively). In contrast, however, rapid addition of two therapeutics at t = 0, yielding the same final, maximum drug concentration achieved by incremental addition above, triggers significant collapse of the leading edge subpopulation within 5 min. Specifically, rapid addition of ibuprofen or acetaminophen decreased the number cells with extended leading edges 70 ± 5% (p < 0.002) and 26 ± 6%, (p < 0.03), respectively, followed by gradual recovery within 2 hrs.

### Population analysis of the effects of rapid drug addition on the leading edge pseudopod

**Fig 4:**
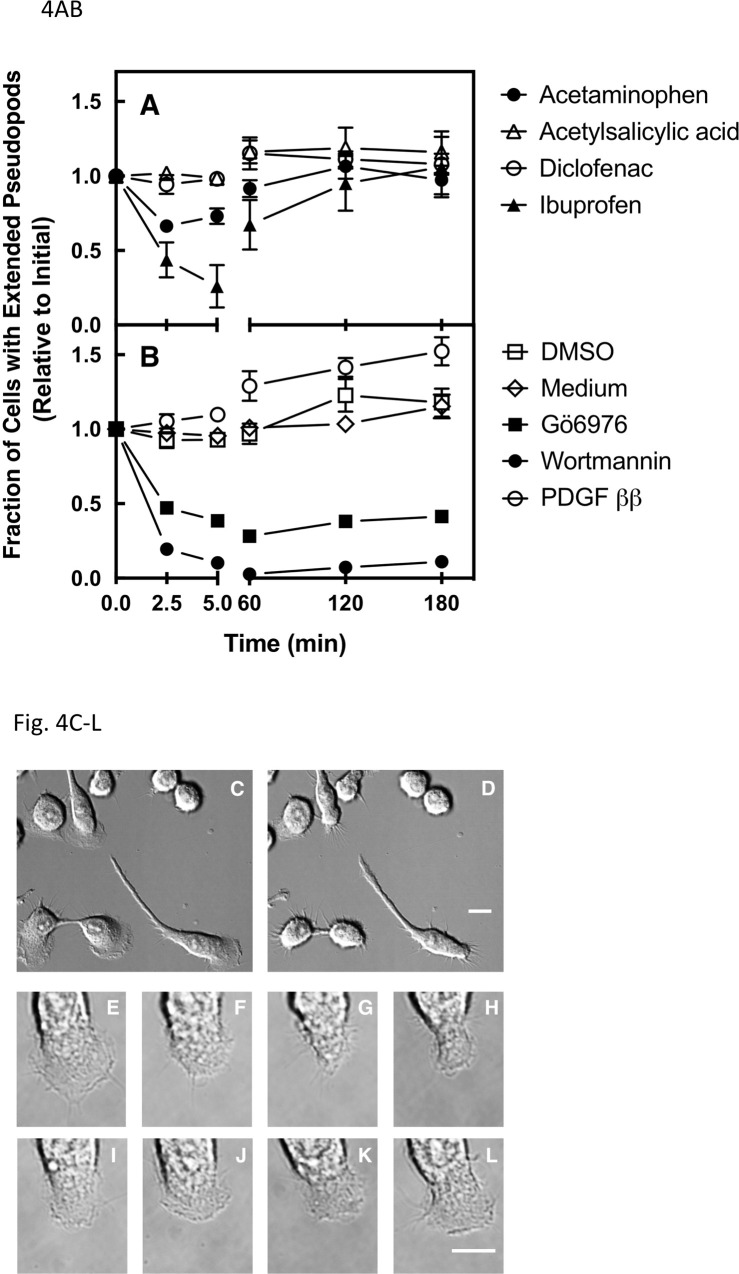
Effects of rapid drug addition on populations of polarized macrophages. RAW macrophages were plated on glass at 37°C in the absence of an attractant gradient (see Methods), then images of cell populations containing large numbers of spontaneously polarized cells with extended leading edge pseudopods were acquired as described in Methods. Drugs were added rapidly at t = 0 to the total concentration indicated in Fig 2. Shown are timecourses following drug addition illustrating the changing number of cells in the full population that display extended leading edge pseudopods, normalized to the initial number prior to drug addition. *Panels A and B* summarize findings for the four therapeutic drugs (A) and for the three control drugs and two carriers (B), respectively. Data collected for multiple population measurements was averaged, yielding the illustrated mean timecourse for each treatment. Error bars are SEM for N = 6 experiments carried out on at least five days for experimental treatments, or at least two days for control treatments. Error bars smaller than their data point symbol are not visible. *Panels C through L* show representative cell images in these studies, as follows. (C,D) Small subsection of a DICM super-image used to quantify the number of cells with extended leading edge pseudopods in a population before and 5 min after ibuprofen addition, respectively. Dividing cells are occasionally observed as illustrated by the pair at lower left. (E-L) Expanded sequential images showing the leading edge pseudopod of a single polarized macrophage within a super-image, illustrating temporary pseudopod collapse after rapid ibuprofen addition at t = 0, followed by pseudopod adaptation and recovery. Panels E-L correspond to timepoints 0, 2.5, 5, 9, 30, 60, 120, 180 min after ibuprofen addition, respectively. Scale bars indicate 10 μm.

As illustrated in Fig 3, gradual and rapid addition of ibuprofen or acetaminophen yields dramatically different effects on the leading edge pseudopod, thus the effects of rapid drug addition were examined more carefully. Fig 4 compares timecourses observed following rapid addition of the four therapeutic drugs, as well as three control drugs for which the effects of rapid addition are well documented in the literature. Fig 4A summarizes the effects of the therapeutic drugs, each added rapidly at t = 0 to their standard peak clinical concentration (Fig 2), on the relative number of cells possessing extended leading edge pseudopods per unit observation area. Rapid acetylsalicylic acid or diclofenac addition had no significant effect on the number of cells with extended pseudopods over a 3 hour observation period, yielding timecourses indistinguishable from that of control vehicle addition (Fig 4A and 4B). In contrast, within 5 min of addition, ibuprofen and acetaminophen each triggered significant reduction of the number of cells with extended pseudopods (decreased 74 ± 14%, p < 0.001 and 27 ± 5%, p = 0.008, respectively; see also Figs 3A and 4C–4L and S2. Again, as observed in Fig 3A, within 2 hrs of ibuprofen or acetaminophen addition the number of cells with extended pseudopods returned to the initial level, indicating that the macrophage population recovered from each drug perturbation by an unidentified mechanism (Fig 4A; see also Figs 4E–4L and S3).

Figs 4B and S2 show the effects of the three control drugs, rapidly added at t = 0 to their standard working concentration (Fig 2), on the normalized number of pseudopod-possessing cells in the RAW macrophage population. Global addition of the pathway activator (PDGF ββ), a known macrophage chemoattractant, triggered a significant increase (by 52 ± 9%, p = 0.002) in the number of pseudopod-possessing cells within 3 hours. At the other extreme, addition of a control pathway inhibitor (either wortmannin or Gö6976 to inhibit PI3K or PKC, respectively) triggered a major decrease (by 90 ± 1%, p < 0.001 or 62 ± 3%, p < 0.001 respectively) in the number of observed pseudopods within 5 min, with little or no recovery during the 3 hour observation period as expected for standard inhibitors of essential components of the leading edge pathway.

### Single cell analysis of the effects of rapid drug addition on the leading edge pseudopod and its PIP_3_ signal

To further investigate the effects of rapid drug addition on leading edge pseudopod morphology and its signature PIP_3_ lipid signal, single cell imaging was carried out. Such single cell measurements provide enhanced spatial and time resolution of within the 5 min observation period following drug addition, but observations could not be extended over a period of hours for technical reasons (on this timescale, even slowly moving cells plated on glass must be imaged periodically to maintain single cell identity, which bleaches the AKT-PH-mRFP PIP_3_ sensor). For each drug or control treatment, an average timecourse was obtained based on observations of 17–32 individual, highly polarized RAW macrophages with extended, ruffling leading edge pseudopods, as illustrated above in Fig 1A and 1C.

**Fig 5:**
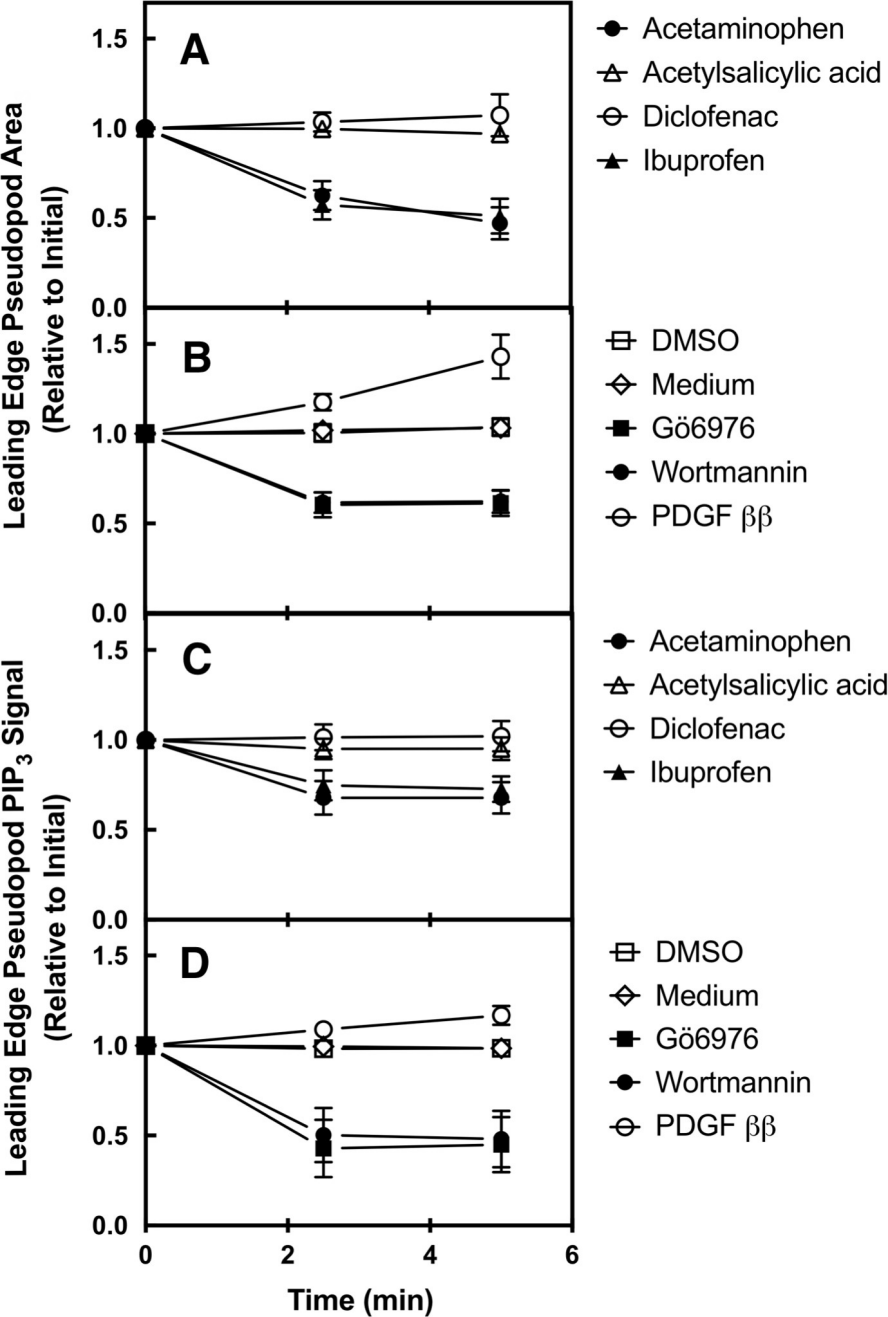
Effects of rapid drug addition on polarized macrophages: Single cell analysis. RAW macrophages were plated on glass at 37°C in the absence of an attractant gradient. Individual, spontaneously polarized cells exhibiting extended, ruffling, leading edge pseudopods were imaged for 5 min as previously described ([26] and Methods). Drugs were added rapidly at t = 0 to their standard total concentration (Fig 2). *Panels A and C* show the timecourses of leading edge pseudopod area loss following the addition of the four therapeutic drugs (A), and the three control drugs or media controls (C). *Panels B and D* show the timecourses of leading edge pseudopod PIP_3_ loss following the addition of the four therapeutic drugs (B), and the three control drugs or media controls (D). All timecourses were quantified as previously described [26]. Briefly, a box of uniform dimensions and placement was used to define the leading edge region of each cell, then the leading edge area or fluorescence was quantified at the indicated timepoints. Note these parameters do not decay to zero, even for the strongest pseudopod inhibitors wortmannin and Gö6976, because the box contains both the pseudopod and the tip of the cell body at t = 0. Thus, the final timepoints at t = 5 min show the loss of area and fluorescence due to the pseudopod collapse, but retain the signals arising from the cell body remaining within the box, respectively. Error bars are SEM for N = 17 to 32 cells over at least three experiments on at least two different days. Error bars smaller than their data point symbol are not visible.

Fig 5 displays average single cell timecourses following rapid addition at t = 0 of the four therapeutic drugs to their standard total concentrations (Fig 2). Rapid addition at t = 0 of acetylsalicylic acid or diclofenac has little or no detectable effect on the pseudopod area (Fig 5A) and PIP_3_ levels (Fig 5C). By contrast, rapid addition of ibuprofen or acetaminophen triggers significant loss of pseudopod area (decreased 49 ± 10%, p < 0.001, or 53 ± 9%, p < 0.001, respectively; Fig 5A) and significant loss of pseudopod PIP_3_ (decreased 27 ± 7%, p < 0.001, or 32 ± 9%, p < 0.001, respectively; Fig 5C) within 5 min. The observation that rapid ibuprofen or acetaminophen addition triggers both collapse of pseudopod area and decreased PIP_3_ levels is consistent with pseudopod destabilization via inhibition of the leading edge signaling pathway.

Fig 5 also summarizes the effects of the three control drugs on the leading edge pseudopod. Addition of the known pathway activator (PDGF ββ) led to a significant pseudopod area expansion (increased 43 ± 12%, p < 0.001) and increased PIP_3_ levels (by 17 ± 5%, p < 0.001) during the 5 min observation period (Fig 5B and 5D). By contrast, the two control pathway inhibitors wortmannin and Gö6976 each triggered significant losses of both the pseudopod area (decreased 38 ± 6%, p < 0.001 and 39 ± 7%, p < 0.001, respectively) and the PIP_3_ signal (decreased 52 ± 16%, p < 0.001 and 55 ± 15%, p < 0.001, respectively) (Fig 5B and 5D). The figure also shows that carriers alone had no detectable effect on the pseudopod.

The observation that a subset of the tested compounds significantly perturb pseudopod area and PIP_3_ levels in single polarized cells raises the possibility these drugs may directly and specifically modulate the enzyme activity of PI3K lipid kinase. Control studies summarized in supplemental S4 Fig confirm that wortmannin, a known irreversible PI3K inhibitor, directly blocks the PIP_3_ production of purified PI3K *in vitro* as previously observed [26, 81]. In contrast, the other drugs have little or no direct effect on the PIP_3_ production of purified PI3K, indicating that only wortmannin directly inhibits PI3K activity *in vitro*.

### Mass spectrometry analysis of drug levels during the three hour cell observation period

**Fig 6:**
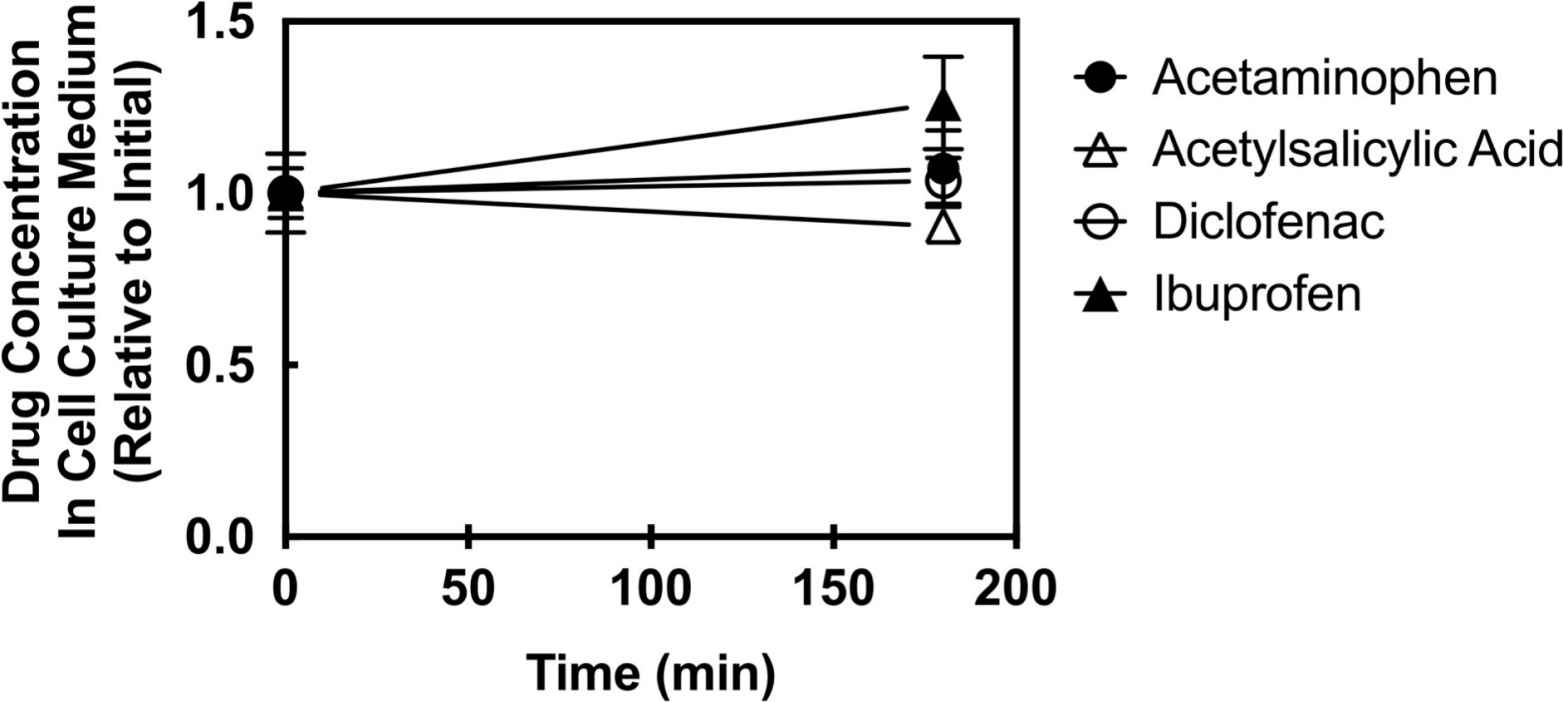
Stability of drug concentrations in cell culture after rapid drug addition. Each of the four therapeutic drugs was analyzed in standard population imaging experiments (Fig 4) to ascertain the stability of the total drug concentration in the media surrounding the cells. Aliquots of media were removed from cell imaging wells immediately after rapid drug addition at t = 0 to the total concentration indicated in Fig 2, and again at t = 3 hr after completion of imaging, then snap frozen in liquid N_2_. Later, the samples were prepared for quantitation with addition of internal standards (Methods), then quantitative mass spectrometry was carried out to compare the initial and final total drug concentrations. Error bars are SD for n = 3 (acetylsalicylic acid, diclofenac) or 6 (ibuprofen, acetaminophen).

The finding that two therapeutic drugs had no detectable effect on the leading edge pseudopod, while two other therapeutic drugs triggered an initial pseudopod collapse followed by recovery, raised the possibility that drugs might be unstable in the media. Quantitative mass spectrometry analysis was carried out to ascertain whether each of the four target drugs was present at the same total concentration during three hours in culture. Fig 6 shows that each of the four therapeutic drugs was present in the cell culture media at the intended clinical concentration at the first timepoint after addition, and exhibited relatively little change during the three hour incubation. Thus, each of the four drugs remained at an approximately fixed, standard clinical concentration during the timecourses described in this study.

## Discussion

The present findings reveal that gradual exposure of cultured RAW macrophages to four representative therapeutic drugs (acetylsalicylic acid, diclofenac, ibuprofen, acetaminophen) has little or no effect on the native morphology of the leading edge sensory pseudopod. Thus, when the concentration of each drug is increased in four equal, stepwise increments up to its standard clinical total concentration (Fig 2) over a period of 90 min, no significant loss of polarized cells with extended pseudopods is detected. These findings are consistent with the well-documented safety of these therapeutics in the clinic, where standard oral dosage also yields a gradually increasing drug level in the plasma up to the total concentration employed herein (Fig 2) within ~90 min [79, 80, 82–84].

Strikingly different results are observed, however, when the same four therapeutic drugs (acetylsalicylic acid, diclofenac, ibuprofen, acetaminophen) and three control drugs (PDGF, Gö6976, wortmannin) are added rapidly to cultured RAW macrophages. Such rapid drug addition, in one step at t = 0 that raises the drug up to its standard total concentration (Fig 2) within 10 sec, yields at least four operational classes of drug effects on the leading edge pseudopod and its signature PIP_3_ lipid signal. (i) Non-perturbing rapid exposures yield little or no effect on pseudopod area, PIP_3_ levels and stability (as observed for acetylsalicylic acid, diclofenac). (ii) Adaptive rapid exposures trigger temporary collapse of the pseudopod followed by recovery (observed for ibuprofen, acetaminophen). (iii) Disruptive rapid drug exposures yield long-term pseudopod collapse with little or no adaptation over the 3 hour observation period (Gö6976, wortmannin). (iv) Activating exposures yield pseudopod expansion and increased cell polarization (PDGF).

To analyze the molecular mechanisms of these four different classes, it is useful to begin by noting the significant drug buffering capacities of human plasma and cell culture media. This buffering capacity is dominated by drug binding to the highly homologous proteins human serum albumin (HSA) and bovine serum albumin (BSA), respectively. Both HSA and BSA possess two high affinity drug binding sites and multiple low affinity sites [49, 85–87]. As a result, in human plasma the four well-studied therapeutic drugs employed herein each show significant binding to HSA at their standard total concentrations, ranging from 24 to 99% bound (Fig 2). Drug binding to serum albumins is rapid, often on the msec timescale depending on the drug and albumin concentrations. While the drug binding properties of HSA and BSA are extremely similar [87, 88], their concentrations are quite different in human plasma (HSA, 500–800 μM) [89, 90] vs cell culture medium (BSA, 30–50 μM; provided by 10% FBS containing 300–500 μM BSA (Methods)). As a representative example, we can estimate the rate of ibuprofen binding to albumin binding sites under these conditions since its HSA equilibrium association constant (K_a_ = 2.3 x 10^6^ M^-1^) and off-rate constant (k_off_ = 0.055 sec^-1^) are known [87, 91]. Assuming the standard total clinical concentration of ibuprofen (240 μM) and the relevant HSA (~500 μM) or BSA (~40 μM) concentration, the initial on-rate for drug binding to protein is ~10 mM per sec or ~1 mM per sec in human plasma or cell culture media, respectively. It follows that the binding of 240 μM ibuprofen to BSA in cultured cell experiments will occur in less than a second, or during the 10 sec mixing time of our drug addition protocol.

Two mechanisms are possible for the lack of pseudopod perturbation observed upon rapid addition of the non-perturbing drugs acetylsalicylic acid and diclofenac: either the free drug could be non-perturbing, or serum albumin buffering in the media could ensure that little free drug is available for cell binding. For acetylsalicylic acid, the high total drug concentration (1.1 mM, Fig 2) relative to the BSA concentration in cell culture media (30–50 μM) ensures that high levels of free drug exceeding 1 mM will be present immediately upon addition to cells. Moreover, mass spec analysis shows the total drug concentration does not change significantly over the 3 hour observation period (Fig 6). It follows that high levels of free acetylsalicylic acid in the millimolar range do not perturb the leading edge pseudopod morphology. For diclofenac, however, the total drug concentration (17 μM) is significantly lower than the BSA concentration in the cell media, and the affinity of this drug for serum albumin is very high (99% bound in human plasma). Thus the free diclofenac concentration in the cell media is likely sub-micromolar, and the lack of pseudopod perturbation could arise from strong drug buffering by BSA that ensures the free drug concentration remains below its unknown pseudopod perturbation threshold.

Two mechanisms are also possible for the temporary collapse of the macrophage pseudopod and its signature PIP_3_ lipid signal upon rapid addition of ibuprofen or acetaminophen. Mechanism (I), which is favored by the available evidence, proposes that the pseudopod is strongly perturbed by appearance of a relatively constant level of free drug triggered by rapid drug addition at t = 0, collapses within 5 min, and then recovers due to cell adaptation to the constant free drug concentration. In contrast, Mechanism (II) proposes that the pseudopod is strongly perturbed by a transient, high local concentration of free drug during addition and mixing that exceeds a drug threshold for pseudopod perturbation. In this model, the transient, high local concentration of drug rapidly decreases as mixing is completed and drug binds to BSA; subsequently, the pseudopod gradually recovers from the initial drug shock simply because the free drug has fallen below its pseudopod perturbation threshold with no contribution from cell adaptation. Multiple lines of evidence support Mechanism (I) for both drugs as follows. The total concentrations of ibuprofen (240 μM) and acetaminophen (170 μM) added to cells are significantly higher than the total BSA concentration in the cell media (30–50 μM), ensuring that substantial free concentrations of drug are present throughout the observation period. Moreover, the affinity of acetaminophen for BSA is low, such that only 24% of the drug is bound even in human plasma where the serum albumin concentration is ~10-fold higher. We cannot formally rule out a version of Mechanism (II) in which rapid addition of the drug stock solution yields a transient spike of high drug concentration; however both mixing and BSA buffering should be complete within ~10 sec and 1 sec, respectively (see Methods and binding rate calculation above). Evidence disfavoring this transient high local concentration model is provided by the observed strong reproducibility with which each drug triggers pseudopod collapse. A mechanism requiring high local drug concentrations during mixing would likely yield variable results between replicates, and between cells in different regions of the same coverslip, with cells close to the addition point more strongly perturbed than distal cells. Spatial heterogeneity is not observed in the large fields of ~1200 cells scanned for population studies (Figs 3 and 4 and S1 Fig), and reproducibility is high in studies monitoring 17–32 single cells (Fig 5). To fully exclude the high local concentration model, future studies will compare the addition of drugs as a 100x stock solution and as a 2x stock solution in cell media. (Such a comparison was planned but prevented by the COVID 19 pandemic and lab shut-down).

The cellular mechanism(s) by which rapid addition of ibuprofen or acetaminophen triggers the initial collapse of leading edge pseudopod and PIP_3_ levels remain unknown, but the findings rule out the possibility that the drugs act as intrinsic PI3K inhibitors since neither blocks PIP_3_ production by purified PI3K in a lipid kinase activity assay (S4 Fig). Thus, the perturbing drugs must directly or indirectly modulate as yet unidentified component(s) of the leading edge signaling pathway, or trigger a nonspecific intracellular perturbation (likely not a pH change, since the drug perturbations are not correlated with drug pKas (Fig 2)).

Similarly, assuming that Mechanism (I) accurately describes ibuprofen and acetaminophen, multiple possibilities exist for the cellular adaptation mechanism(s) that drive pseudopod recovery following the initial drug-triggered collapse. The simplest hypothesis is that the same unknown adaptation mechanism(s) are acting both to drive pseudopod recovery after rapid drug addition, and to prevent pseudopod collapse during slow drug addition. This hypothesis proposes that rapid drug addition overwhelms the adaptation process which is unable to counteract the perturbation in real time, but gradually eliminates the perturbation within 3 hours. In contrast, gradual drug addition allows concurrent gradual adaptation that is able to counteract the drug perturbation in real time. Adaptation does not involve extracellular chemical or enzymatic degradation of drugs in the media surrounding the cultured cells, since mass spectrometry analysis indicates that each drug is stable in the media during the three hour cell incubation (Fig 6). Instead, the adaptation mechanism is hypothesized to be intracellular. Such intracellular adaptation could be provided by the intrinsic adaptation mechanisms of the leading edge chemosensory pathway which, like other sensory pathways, must adapt to changing background stimuli and attractant concentrations as the cell moves through different environments [41, 42, 92, 93]. Other possible adaptive mechanisms could include activation of an intracellular sink, pump, degradation or modification reaction that eliminates free drug in the cytoplasm, but has a negligible effect on the bulk extracellular drug concentration. Such mechanisms have been reported for other drugs (reviewed in [47, 48]).

The mechanisms of the three control drugs are well defined by previous studies. Exposures to the non-clinical control inhibitors wortmannin and Gö6976 are disruptive, yielding long term collapse of the leading edge pseudopod and its PIP_3_ lipid signal. These drugs are known to directly inhibit essential components of the leading edge positive feedback loop (PI3K and PKC, respectively), and their disruption of the leading edge pseudopod upon rapid addition has been previously described [26, 30, 50–54]. Rapid exposure to PDGF yields pseudopod expansion and increased cell polarization, as previously observed and commensurate with its known importance as a strong macrophage attractant *in vitro* and *in vivo* [26, 55].

In closing, the present findings suggest a number of directions for future research. Further studies are needed to determine the molecular mechanisms by which rapid ibuprofen or acetaminophen addition trigger macrophage leading edge pseudopod collapse, and by which the pseudopod adapts (or recovers) from these drug exposures. Given the ~10-fold higher concentrations of albumins in plasma compared to cell culture media, the greater drug buffering capacity of plasma will help protect macrophages and other cell types from drug perturbations, but the sensitivity of the cultured macrophage leading edge pseudopod to drugs makes it an excellent model system for detecting and classifying drugs capable of inhibiting macrophages, either transiently or permanently. Transient macrophage inhibition could be useful in controlling extreme inflammation events, while long-term inhibition that blocks macrophage participation in the innate immune response would be highly detrimental. It will be important to carry out future studies that directly test whether drug-induced pseudopod collapse causes inhibition of macrophage motility and chemotaxis *in vitro* and *in vivo* [94]. More broadly, we hypothesize that the same four classes of drug effects observed herein for cultured macrophages will also be observed for primary macrophages and other leukocytes such as neutrophils which possess similar chemosensory pathways [15, 20, 32, 46], but this prediction also remains to be tested. Finally, the effects of other therapeutic and experimental drugs on the leading edge pseudopod remain to be determined. In short, the present study opens several new research paths with major implications for a basic understanding of drug-induced perturbation and adaptation in leukocyte signaling pathways.

## Supporting information

S1 Fig Imaging a population of cultured RAW macrophages. RAW macrophages were plated on glass at 37°C in the absence of an attractant gradient. Populations of cells were imaged in a large field containing 1200 ± 100 cells, which was scanned as either 6 x 6 (for example (A)) or 8 x 8 individual unit areas that were subsequently stitched together into a superimage (see Methods). The inset (B) shows an expanded view of a region within a single unit area, illustrating the resolution of single cell morphologies ranging from highly polarized cells with extended leading edge pseudopods to rounded, unpolarized cells.
(TIFF)

S2 Fig Short term effects of drugs on the leading edge pseudopod of cultured RAW macrophages. RAW macrophages were plated on glass at 37°C in the absence of an attractant gradient. Individual, spontaneously polarized cells exhibiting extended, leading edge pseudopods were visualized in DICM superimages captured as described in Methods. The left image in each pair was captured at t = 0, then the indicated drug was rapidly added to the total concentration indicated in Fig 2 and right image was captured at t = 5 min.
(TIFF)

S3 Fig Long term recovery of the leading edge pseudopod of cultured RAW macrophages following initial perturbation by adaptive drugs. RAW macrophages were plated on glass at 37°C in the absence of an attractant gradient. Individual, spontaneously polarized cells exhibiting extended, leading edge pseudopods were visualized in DICM superimages captured as described in Methods. An image was captured at t = 0, then the indicated drug was rapidly added to the total concentration indicated in Fig 2 and subsequent images were captured at 2.5, 5, and 60 min.
(TIFF)

S4 Fig Effect of drugs on the lipid kinase activity of purified PI3K in an *in vitro* PIP_3_ production assay. A single molecule TIRFM assay was employed to measure the specific kinase activity of purified PI3K molecules on a supported lipid bilayer by counting the number of PI3K molecules, as well as the number of product PIP_3_ molecules they produce as previously described in detail [20–23]. This assay utilizes physiological concentrations of class I PI3Ka and saturating concentrations of fluorescently-labeled GRP-PH domain, a high-affinity PIP_3_ product lipid binder, in order to count each product lipid produced. Drug is added to therapeutic dose identified in Fig 2 prior to the start of the assay. Enzyme rates are calculated as the slope of product lipid created vs. time. Bars indicate the lipid kinase activity of PI3K in the presence of the indicated drug. Error bars are SD where n = 3 for acetylsalicylic acid and diclofenac, or n = 6 for ibuprofen and acetaminophen.
(TIFF)

## Abbreviations

PI3K: phosphoinositide-3-kinase
PKC: protein kinase C
PIP_**3**_: phosphatidylinositol (3,4,5)-trisphosphate
COX: cyclooxygenase
NSAID: nonsteroidal anti-inflammatory drug
PDGF ββ: platelet-derived growth factor ββ homodimer
DICM: differential interference contrast microscopy
TIRFM: total internal reflection fluorescence microscopy
