# Supplementary figures and images for: Rapid exposure of macrophages to drugs resolves four classes of effects on the leading edge sensory pseudopod: Non-perturbing, adaptive, disruptive, and activating
Source: PLoS One. 2020 May 29;15(5):e0233012. doi: 10.1371/journal.pone.0233012 (PMC7259666; doi:10.1371/journal.pone.0233012)

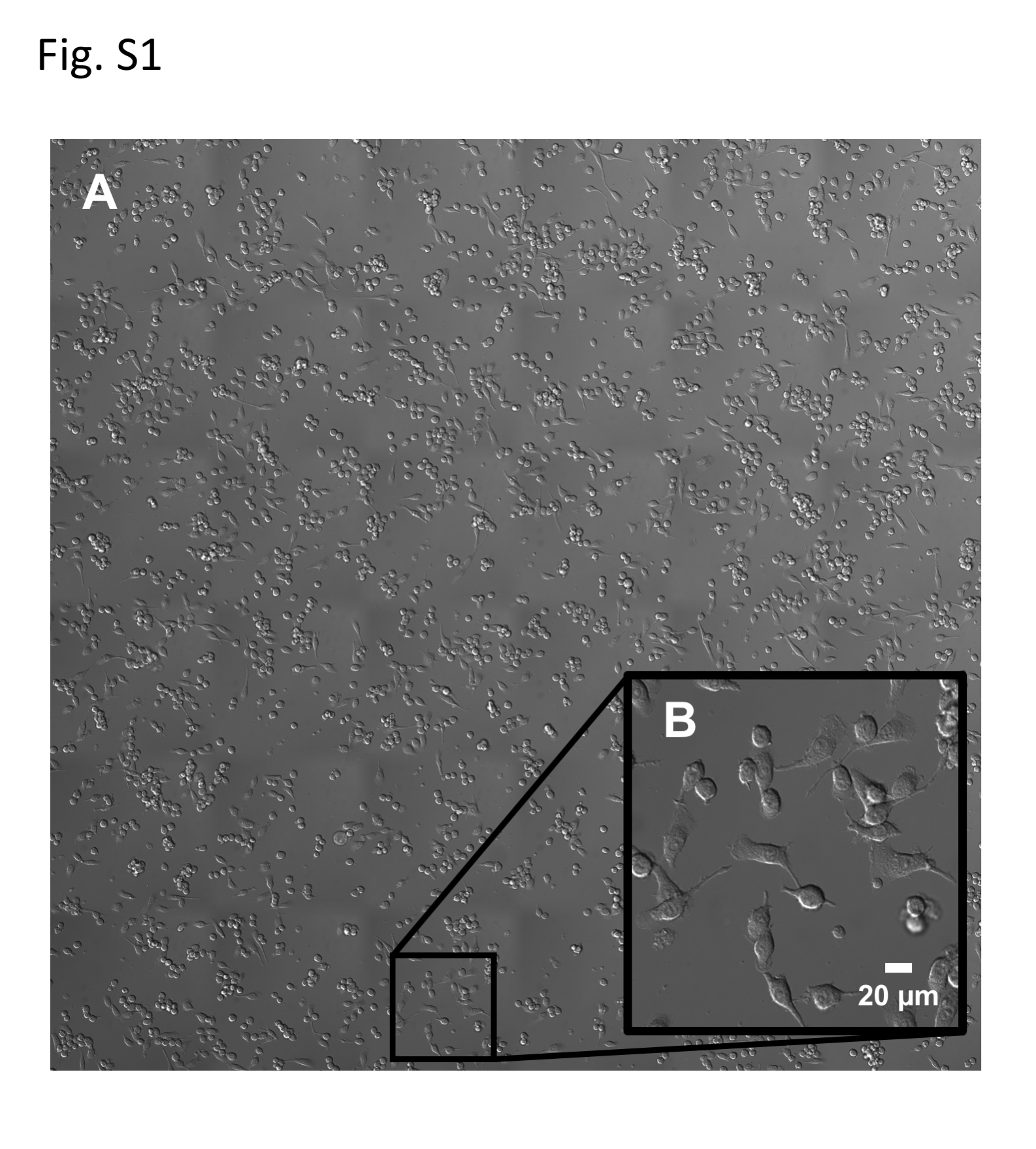

Supplement: S1 Fig — RAW macrophages were plated on glass at 37°C in the absence of an attractant gradient. Populations of cells were imaged in a large field containing 1200 ± 100 cells, which was scanned as either 6 x 6 (for example (A)) or 8 x 8 individual unit areas that were subsequently stitched together into a superimage (see Methods). The inset (B) shows an expanded view of a region within a single unit area, illustrating the resolution of single cell morphologies ranging from highly polarized cells with extended leading edge pseudopods to rounded, unpolarized cells. (TIFF) [file pone.0233012.s001.tiff]

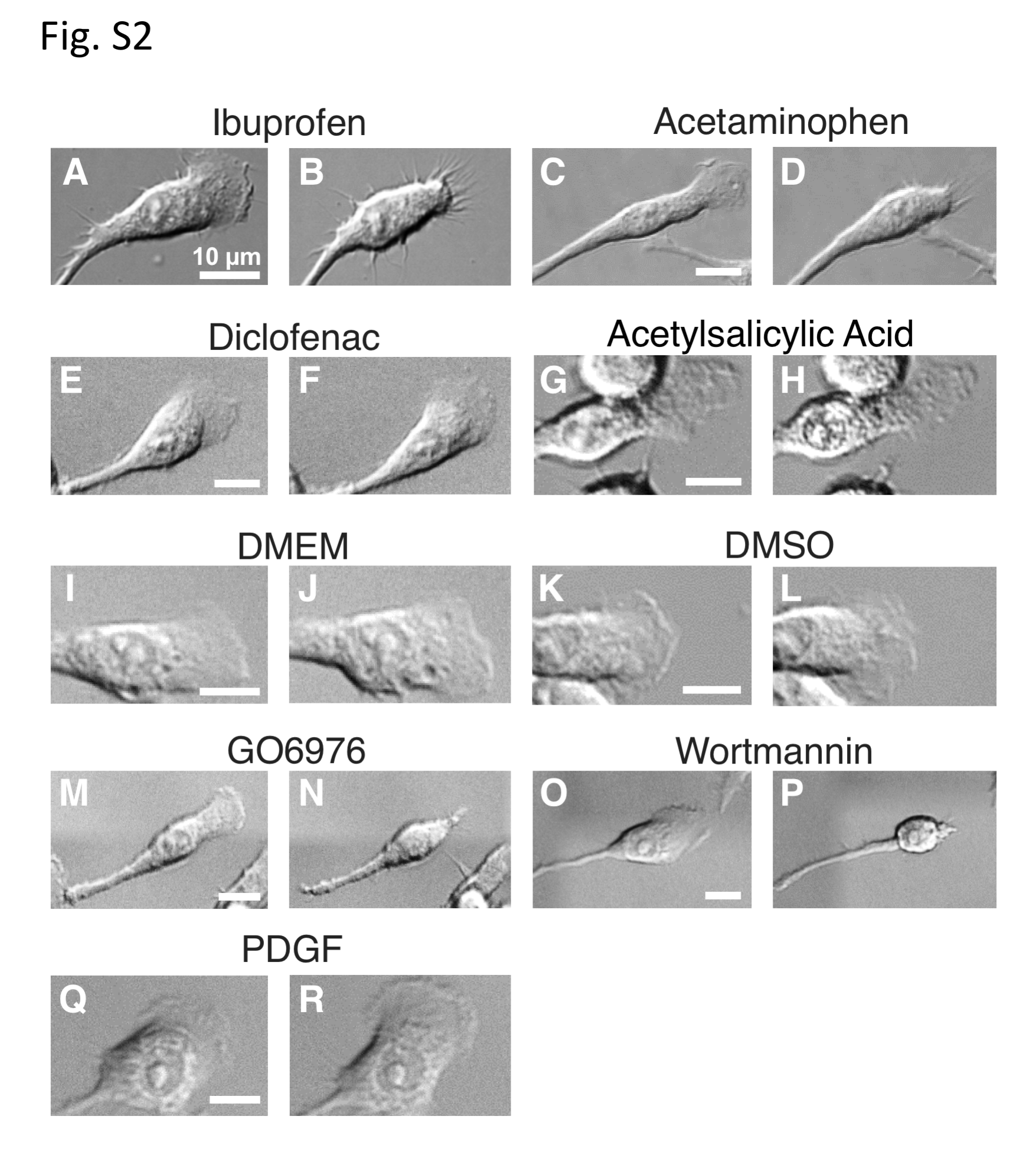

Supplement: S2 Fig — RAW macrophages were plated on glass at 37°C in the absence of an attractant gradient. Individual, spontaneously polarized cells exhibiting extended, leading edge pseudopods were visualized in DICM superimages captured as described in Methods. The left image in each pair was captured at t = 0, then the indicated drug was rapidly added to the total concentration indicated in Fig 2 and right image was captured at t = 5 min. (TIFF) [file pone.0233012.s002.tiff]

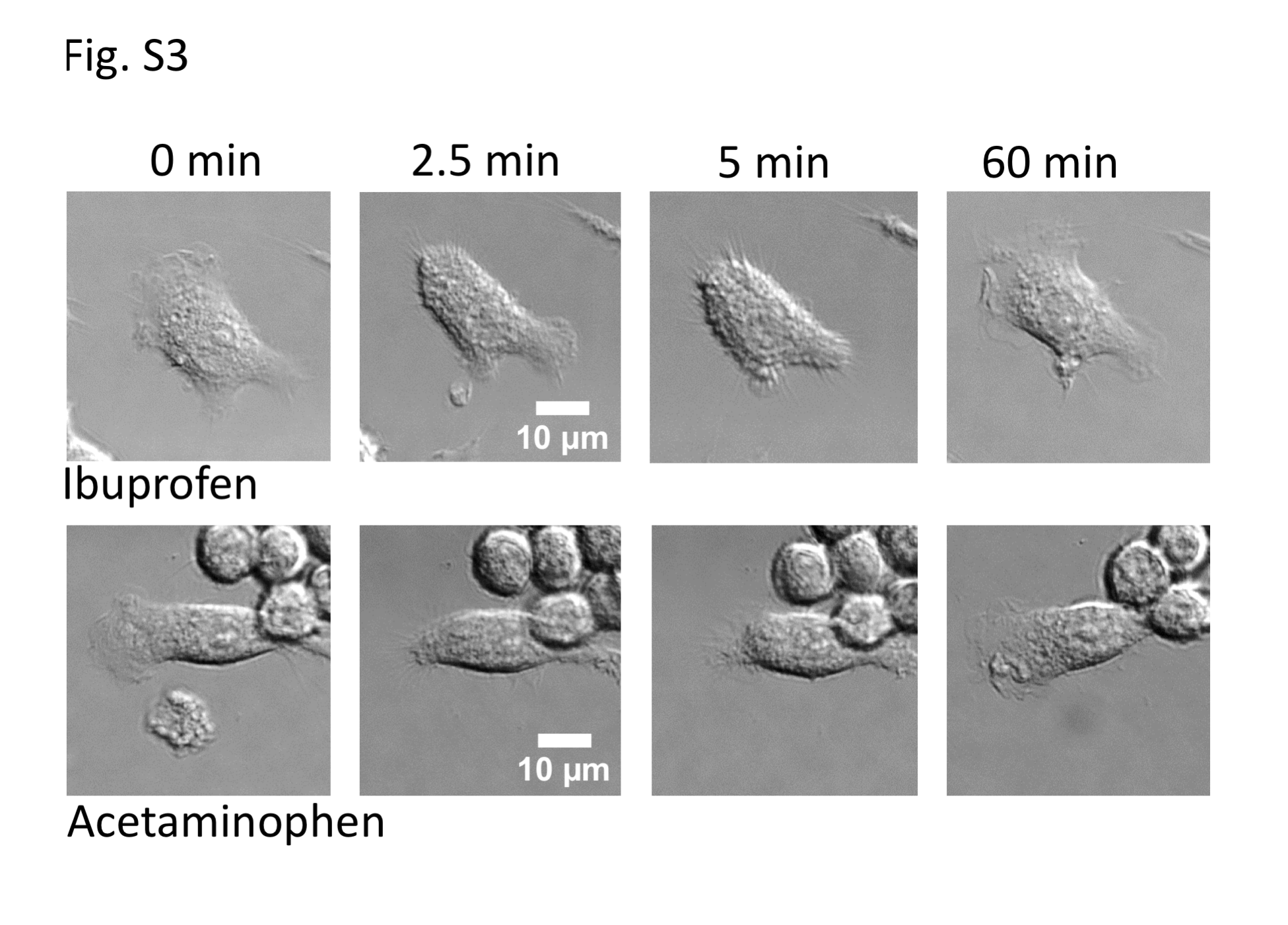

Supplement: S3 Fig — RAW macrophages were plated on glass at 37°C in the absence of an attractant gradient. Individual, spontaneously polarized cells exhibiting extended, leading edge pseudopods were visualized in DICM superimages captured as described in Methods. An image was captured at t = 0, then the indicated drug was rapidly added to the total concentration indicated in Fig 2 and subsequent images were captured at 2.5, 5, and 60 min. (TIFF) [file pone.0233012.s003.tiff]

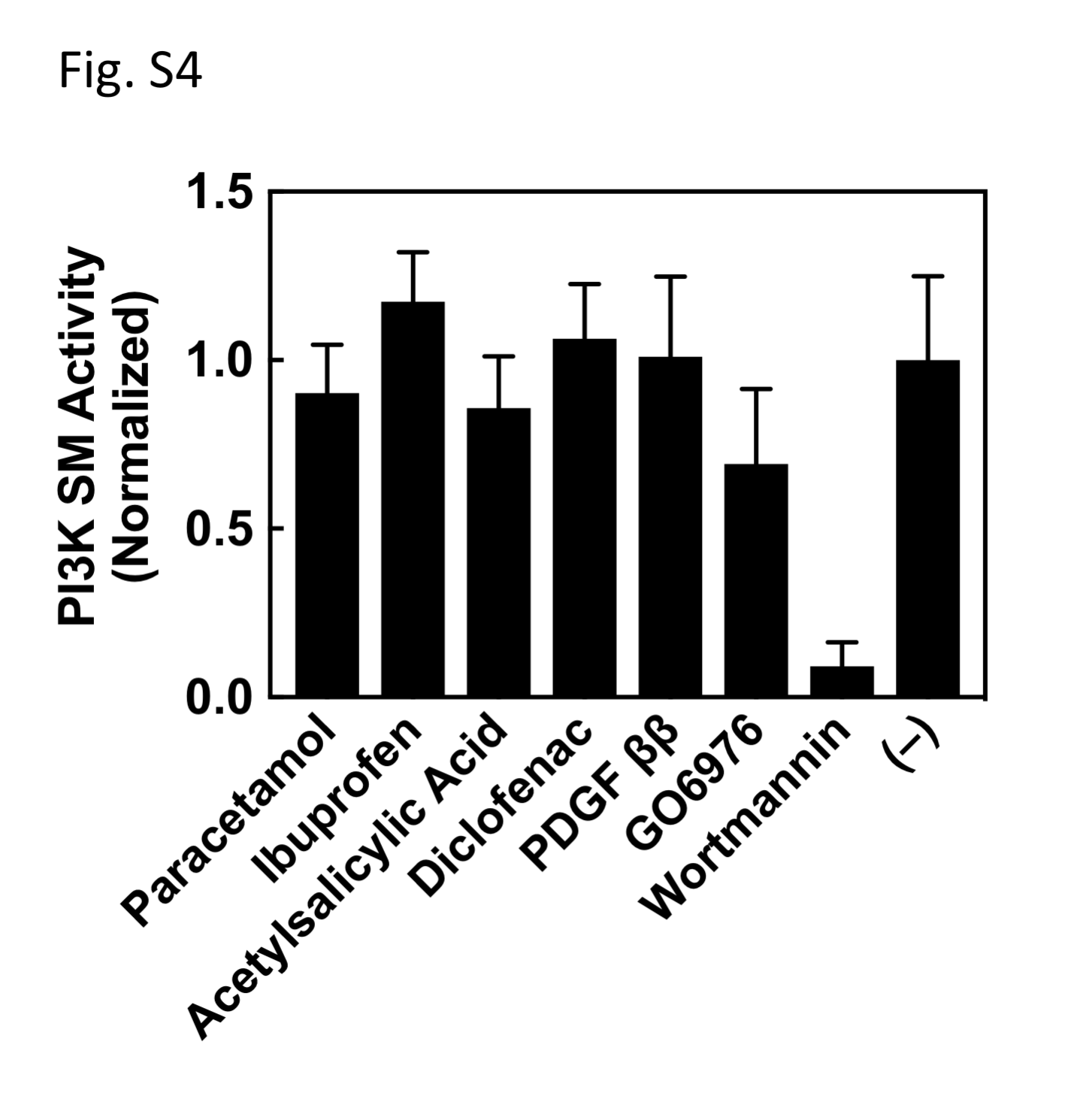

Supplement: S4 Fig — A single molecule TIRFM assay was employed to measure the specific kinase activity of purified PI3K molecules on a supported lipid bilayer by counting the number of PI3K molecules, as well as the number of product PIP3 molecules they produce as previously described in detail [20–23]. This assay utilizes physiological concentrations of class I PI3Ka and saturating concentrations of fluorescently-labeled GRP-PH domain, a high-affinity PIP3 product lipid binder, in order to count each product lipid produced. Drug is added to therapeutic dose identified in Fig 2 prior to the start of the assay. Enzyme rates are calculated as the slope of product lipid created vs. time. Bars indicate the lipid kinase activity of PI3K in the presence of the indicated drug. Error bars are SD where n = 3 for acetylsalicylic acid and diclofenac, or n = 6 for ibuprofen and acetaminophen. (TIFF) [file pone.0233012.s004.tiff]
